# Factorial design-assisted reverse phase HPLC–UV approach for the concurrent estimation of cetirizine and azelastine in aqueous humor

**DOI:** 10.1038/s41598-022-26774-y

**Published:** 2022-12-27

**Authors:** Walaa Nabil Abd-AlGhafar, Fatma Ahmed Aly, Zeinab Awad Sheribah, Samar Saad

**Affiliations:** Pharmaceutical Analytical Chemistry Department, Faculty of Pharmacy, Mansoura University, Mansoura, 35516 Egypt

**Keywords:** Analytical chemistry, Medical and clinical diagnostics

## Abstract

A new analytical quality by design-assisted HPLC–UV approach is presented, for the first time, for the concurrent determination of cetirizine (CTZ) and azelastine (AZE) in raw materials, commercial eye drops and aqueous humor. The two drugs are co-administered as eye drops in severe ocular allergies. A 2^3^ full factorial design was adopted for the chromatographic optimization to ensure the best analytical performance and reliability, as well as to save time, effort and solvent consumption. The parameters, including pH, acetonitrile ratio, and flow rate, were selected as independent factors. The responses analyzed were resolution and tailing of peaks. The separation was achieved through isocratic elution on C8 column with mobile phase made up of acetonitrile: 0.3% triethylamine of pH 5 (60:40 v/v) at a flow rate of 1.2 mL min^−1^ and detection at 216 nm. The elution time was less than 6 min. The approach was fully validated in accordance with International Council for Harmonization (ICH) guidelines. Good linearity was achieved over the concentration ranges of 1.0–30 and 0.5–10 µg mL^−1^ with limits of detection of 0.310 and 0.158 µg mL^−1^ and limits of quantification of 0.940 and 0.479 µg mL^−1^ for CTZ and AZE, respectively, with correlation coefficients of 0.9998. The intra- and inter-day precisions were lower than 2%. The good sensitivity of the approach permits the analysis of CTZ and AZE in spiked aqueous humor with mean percentage recoveries of 100.93 ± 1.42 and 100.11 ± 1.55, respectively. The statistical comparison between results of the developed method and the comparison method revealed no differences, indicating the accuracy of the method.

## Introduction

Quality by design has emerged as a cutting-edge systematic approach that produces scientifically sound results. The application of design of experiments (DOE) in chromatographic separation has several merits in optimization and establishment of a robust and rugged method. It is an efficient tool for optimization of the experimental condition to obtain maximum information with lower number of experiments than univariate procedures^1, 2^. This matches the current direction for developing green analytical approaches with less investment, effort and solvents. DOE tests multiple factors simultaneously to obtain a certain response and interaction between them^3^.

Ocular allergy is one of the most common ocular surface diseases that has emerged in the last several decades^4^. It affects approximately 40% of the population globally^5^. It reduces the patient’s productivity at work as well as his overall quality of life^4^. Ocular allergy disease is the inflammation of the conjunctiva, which is caused by an immune system reaction mediated by immunoglobulin-E^6^. It is caused by mold, pollen and environmental stimuli leading to watery and swollen eye, itching and chemosis^7^. Because of the widespread and multifactorial causes of ocular allergy, a combination of therapies is often required to treat the associated signs and symptoms.

**Figure 1:**
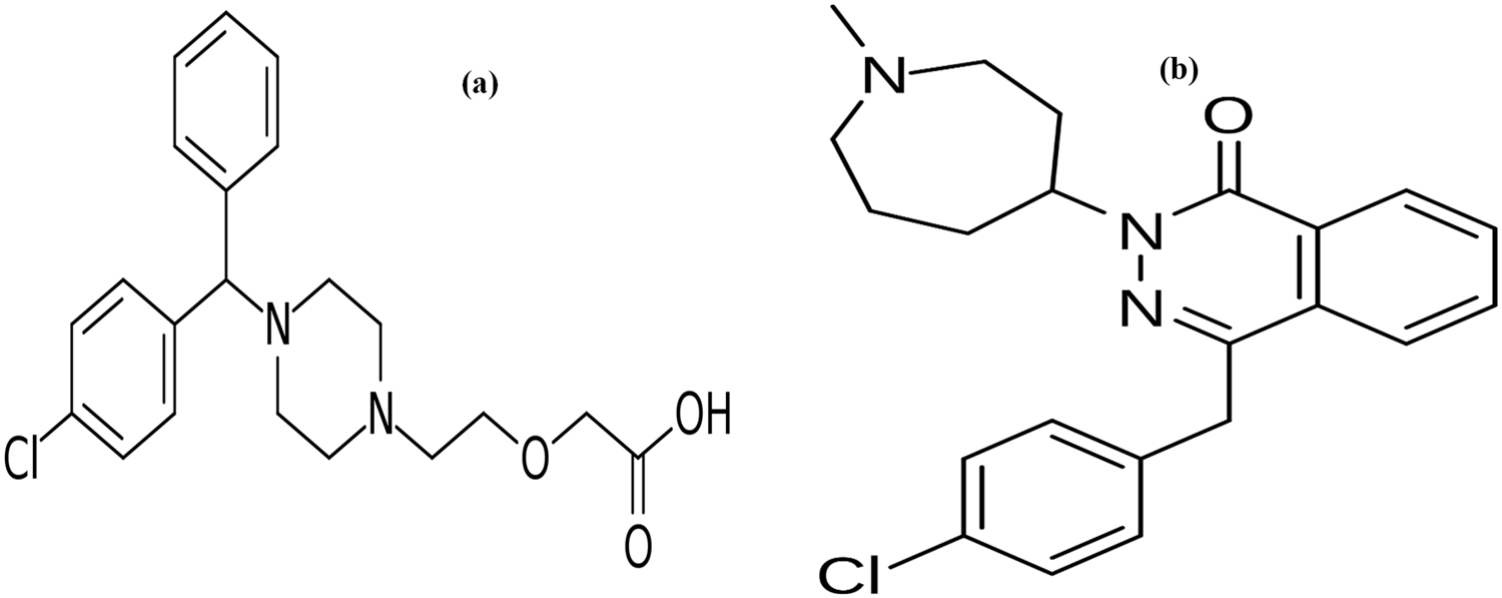
Chemical structure of (**a**) cetirizine and (**b**) azelastine.

CTZ is [(4-chlorophenyl) phenylmethyl]piperazin-1-yl] ethoxy] acetic acid (Fig. 1a)^8^. It is a second-generation antihistamine which is widely used in the comprehensive management of allergic conjunctivitis^9^. BP^8^ stated non-aqueous potentiometric titration with sodium hydroxide, whilst USP^10^ described high performance liquid chromatography (HPLC) procedure for CTZ estimation. Various analytical techniques have been developed for the determination of CTZ either in pharmaceutical preparations, human plasma or aqueous humor as HPLC^11–16^, capillary electrophoresis^17, 18^, spectrofluorimetry^19–23^ and spectrophotometry^24, 25^.

AZE is 4-(4-chlorobenzyl)-2- [-1-methylhexahydro-1H-azepin-4-yl] phthalazin-1(2H)-one (Fig. 1b)^8^. It is a mast cell stabilizer applied topically to treat the symptoms of allergic conjunctivitis^26^. It was determined by non aqueous titration with 0.1 N perchloric acid potentiometrically in both BP^8^ and USP^10^. Different articles have been reported in the literature for the determination of AZE either in dosage form, human plasma or aqueous humor. These articles include: HPLC^27–31^, HPTLC^32, 33^, capillary electrophoresis^34^, electrochemical analysis^35, 36^, NMR^37^, spectrofluorimetry^22, 38–40^ and spectrophotometry^41–43^.

Topical antihistamines and mast cell stabilizers are frequently co-administered for the relief of ocular allergies^44^. CTZ as an antihistamine blocks H1 receptors, providing immediate relief before AZE starts to inhibit mast cell degranulation and histamine release. So, adding AZE can increase the efficacy of CTZ^44^.

Reviewing the literature showed that only one research was conducted in our previous lab for the simultaneous quantification of CTZ and AZE using synchronous spectrofluorimetry^22^. It has narrow linearity ranges for both drugs (0.1–2.0 µg mL^−1^ for both). Sulfuric acid was used in an attempt to improve the sensitivity. On the other hand, the proposed approach avoids the analysis in such a corrosive environment (pH less than 2). The reported HPLC^11^ for CTZ assay used gradient elution and mass spectrometry, which is not available in most laboratories due to its high cost. Our procedure had a shorter retention time for both drugs than the reported HPLC procedures ^12, 13, 16, 27, 30^. The developed HPLC procedure, whose mobile phase is made up of only two components, is much simpler than other documented HPLC procedures^14, 15, 30, 31^, whose mobile phases are made up of three or more components, therefore requiring more time for preconditioning. Also, our procedure had a lower LOQ for both drugs than the documented procedures^13–16, 28, 29^. All of these previous methods were developed by changing one factor at a time, which has assured to be time-consuming, expensive, does not fix errors, and may yield unpredictable responses^45^. Up till now, there has been no described HPLC procedure for the quantification of either CTZ or AZE alone in aqueous humor or the simultaneous quantification of both.

HPLC is currently considered the most widely used analytical technique in the pharmaceutical industry and in the analysis of pharmaceuticals in aqueous humor due to its availability, reproducibility and diversity of applications^46^. The goal of HPLC drug analysis is to verify the identity of a drug and provide quantitative results, as well as to control the progress of disease therapy^47^. It is characterized by ease of automation, permitting high throughput analysis of numerous samples over a short time, making it suitable for clinical assessment. It rendered the procedure more specific and reproducible than the fluorimetric one.

Accordingly, a new, rapid, selective, accurate and cost-effective analytical HPLC method is developed utilizing a full factorial design for the simultaneous estimation of CTZ and AZE in synthetic mixtures, single ophthalmic formulations and aqueous humor. Being co-administered makes their analysis in aqueous humor clinically important, which is why we conducted this study. The results obtained were auspicious with wider linearity ranges than the previously documented approaches and adequate sensitivity for both drugs.

Eye drops must be administered in the right dose to ensure optimal efficacy and minimize side effects for the patient. Novelty of the suggested HPLC method originates from the simultaneous analysis of both drugs in a complex matrix as aqueous humor for therapeutic drug monitoring, which had never been previously studied. There is a lack of chromatographic techniques for the determination of CTZ in ophthalmic formulations. This research paper includes a detailed investigation concerning their separation efficiency and quantification owing to their clinical impact, which in turn is expected to offer help to analysts caring to analyze the cited drugs. It is superior to the documented methods in completing the separation process in much less time, which in turn saves solvent consumption. It achieves a relatively high sensitivity with good peak shapes. It is also the first time to apply DOE (full factorial design) in the development and optimization of HPLC for either CTZ or AZE assay to give the best possible analytical performance rather than changing one factor at a time. With DOE, method developers are given a comprehensive understanding of the method thanks to specialized domain expertise. Knowledge of critical method parameters and design plots will ensure optimal performance of the method. Changing one factor at a time frequently leads to nonrobust performance^2^. The DOE-based technique simultaneously permits the quick and effective development of reliable methods. This encourages our developed approach to be employed as an efficient, easy and robust analytical tool for routine high-throughput analysis required in research centers and quality control laboratories. Additionally, promoting the approach to be carried out in pharmacokinetic investigations.

## Experimental

### Apparatus and software

A Knauer Azura® chromatograph with 20 µL loop and a Rheodyne injection valve was utilized in the chromatographic separation. It was equipped with P 6.1 L pump, 2.1 L UV detector and D 2.1 S degasser (Berlin, Germany). A consort NV P-901 pH meter (Belgium) was utilized for pH adjustment. DOE was performed with the assistance of Minitab® Statistical Software (release 16, State College, PA, USA).

### Materials and reagents

- CTZ (99.95% purity as certified) and AZE (99.80% purity as certified) bulk drugs were obtained from Apex and European Egyptian Pharmaceuticals Industry, respectively.
- Cetirizine® (1% of CTZ) and Azelast® (0.05% of AZE) eye drops are products of Pharo Pharma (batch no. 5669002) and The Tenth of Ramadan for Pharmaceuticals (batch no. 202792), respectively. Both are purchased from the local pharmacy.
- All the organic solvents utilized in the study were HPLC grade. Acetonitrile and ethanol were attained from Sigma Aldrich (Germany). Methanol was obtained from Tedia (USA).
- TEA and Phosphoric acid were gotten from Sigma Aldrich and RiedeldeHäen, Honeywell Research Chemicals (Germany), respectively.

### Standard solutions

CTZ and AZE stock solutions (100 µg mL^−1^ of each) were made individually in acetonitrile. Working standard solutions of CTZ and AZE were made individually by dilution of the stock solutions with acetonitrile to yield a concentration of 20 µg mL^−1^ of both analytes. Afterwards, dilution was made with the mobile phase to prepare solutions within the concentration ranges (1.0–30.0 µg mL^−1^ for CTZ and 0.5–10.0 µg mL^−1^ for AZE). All the solutions were kept in the refrigerator and wrapped in aluminum foil.

### Chromatographic conditions

The separation was accomplished using C8 column (Hypersil MOS, 5 µm particle size, 150 mm × 4.6 mm). The mobile phase made up of 0.3% TEA: acetonitrile (40:60 v/v, pH 5) and delivered at a flow rate of 1.2 ml min^−1^. UV detection was performed at 216 nm.

### General procedures

#### Construction of the calibration curves

Calibration curves were constructed by dilution of each analyte individually from the corresponding stock and working solutions with the mobile phase into a set of 10.0 mL volumetric flasks. The obtained solutions have a concentration range of 1.0–30.0 µg mL^−1^ for CTZ and 0.5–10.0 µg mL^−1^ for AZE. Each solution was injected in triplicate under the optimum chromatographic conditions. The peak area against the ultimate concentration of each analyte (µg mL^−1^) was plotted. Then, the regression equations were derived.

#### Analysis of CTZ/AZE in laboratory prepared mixtures

A set of laboratory prepared mixtures of CTZ and AZE were made by transferring different aliquots of stock and working solutions of both drugs in different ratios into a set of 10.0 mL volumetric flasks. The described procedure under **“****Construction of the calibration curves****”** was adopted. The concentrations of each analyte were calculated with the aid of the corresponding regression equations.

#### Analysis of CTZ and AZE in ophthalmic formulations

For Cetirizine® (1.0% CTZ): The contents of five bottles of the formulation were mixed and aliquot (equivalent to 20.0 mg) was transferred into 100.0 mL volumetric flask and completed to the mark with acetonitrile. Further dilution was made to prepare working standard solution of 20.0 µg mL^−1^. CTZ was analysed as described under **“****Construction of the calibration curves****”**.

For Azelast® (0.05% AZE): The contents of five bottles were mixed and aliquot (equivalent to 1.0 mg) was transferred into 50.0 mL volumetric flask and completed to the mark with acetonitrile (20 µg mL^−1^). AZE was assayed as explained under **“****Construction of the calibration curves****”**.

#### Analysis of CTZ/AZE in aqueous humor

Artificial aqueous humor was prepared to mimic the chemical composition of the human aqueous humor^48^. One mL of it was moved into 10.0 mL volumetric flasks, followed by adding different aliquots of both CTZ and AZE stock and working solutions containing (10–300 and 5–100 µg of CTZ and AZE, respectively). Implement the procedure under **“****Construction of the calibration curves****”**.

## Results and discussion

### Method optimization

Optimization of HPLC method is a complex process that necessitates the simultaneous modification of numerous variables to achieve the desired separation. DOE has recently been employed in order to improve separation quality and reduce the number of trials during the optimization phase^3^. Preliminary experiments should be conducted before applying a factorial design to test the feasibility of the experimental design. These experiments include:

### Choice of column

Three columns were tried in the experiment including:

1. ShimPack CLC-cyanopropyl (150 × 4.6 mm, 5 µm).
2. HyperClone™ ODS C18 (150 × 4.6 mm, 5 µm).
3. HyperClone™ MOS C8 (150 × 4.6 mm, 5 µm).

The last one was the most suitable regarding analysis time and resolution of peaks. Overlapped peaks were obtained using the rest of the listed columns.

### Selection of suitable wavelength

**Figure 2:**
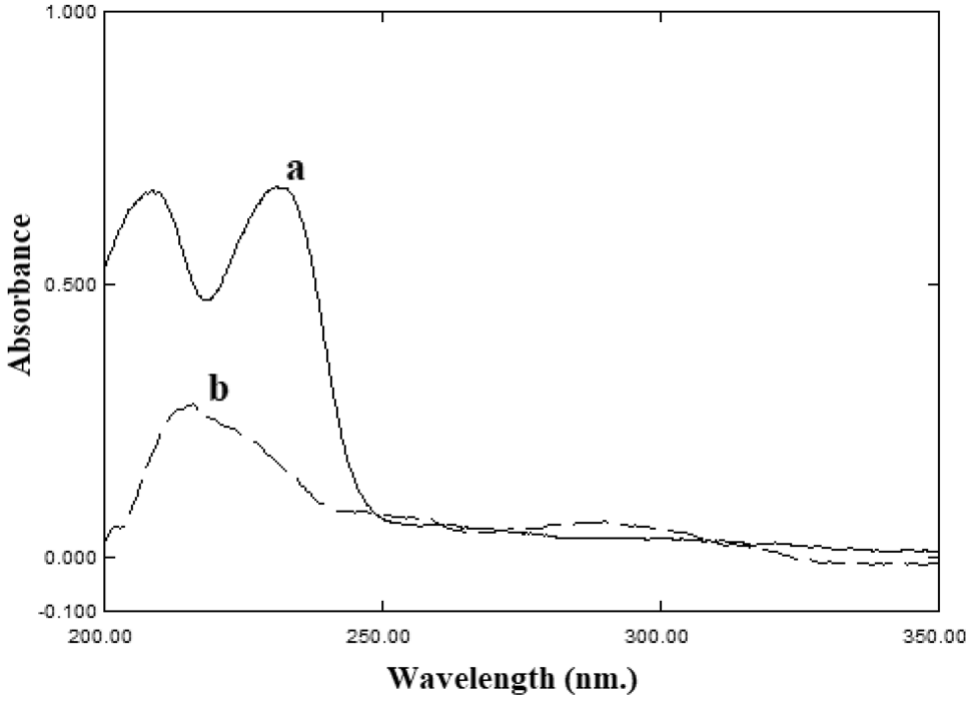
Zero-order absorption spectra of (**a**) 20 µg mL^−1^ CTZ and (**b**) 5 µg mL^−1^ AZE in acetonitrile.

The studied compounds exhibited maxima in their spectra at 209 and 231 nm for CTZ and 216 nm for AZE in acetonitrile (Fig. 2). Therefore, 216 nm was selected as the suitable wavelength with good sensitivity for both compounds.

### Mobile phase composition

#### pH

pKa of CTZ (strongest acidic is 3.58 and strongest basic is 7.74^49^) and pKa of AZE is 9.16^49^. CTZ was delayed as pH decreased due to ionization suppression of the carboxylate anion. On the other hand, AZE would be ionized at low pH values (polar) and less retained. Mobile phase pH range of 4.5–5.0 was chosen for factorial design. In the preliminary trials, upon decreasing pH below 4.5, the resolution between peaks decreased. At pH higher than 5.0, the retention time of AZE increased greatly, with lower sensitivity for both analytes. Log P values of CTZ and AZE are 2.98 and 4.04, respectively^49^. Consequently, CTZ is more polar and eluted the first.

#### Type and ratio of the organic solvent

In the first trials in the screening phase, different organic solvents were tested, including: methanol, ethanol and acetonitrile. Overlapped peaks were obtained upon using methanol. Ethanol led to longer retention times. Therefore, acetonitrile was selected as it gave the best sensitivity, retention times and resolution of peaks. Increasing ratio of more than 60% resulted in poor resolution, while decreasing ratio of less than 50% resulted in longer retention times. Accordingly, ratios between 50–60% of acetonitrile were used as the range for the factorial design.

#### Type of the aqueous component

First of all, different ionic strengths of phosphate buffer with varying pH values using 0.2 M phosphoric acid were tried. But, AZE peak still suffered from unacceptable tailing (AZE tailing factor 2.31). Several attempts were made to overcome this tailing. The aqueous phases tested were: 0.05 M phosphate buffer pH 5 adjusted with either 0.2 M acetic acid or 0.3% TEA, 0.05 M acetate buffer pH 5 adjusted with 0.2 M acetic acid and 0.3% TEA pH 5 adjusted with 0.2 M phosphoric acid. The last one solved the tailing problem of AZE peak. TEA interacts with the silanol group in the column at higher affinity, which protects its interaction with the nitrogen atom of the studied analyte^50^. As a result, it reduces peak tailing. Additionally, this aqueous phase has benefits, including easy preparation and the column does not require too much time for washing after analysis as it does not contain any salt components.

#### Flow rate

A flow rate in the range of 1.0–1.2 mL min^−1^ was chosen for the factorial design. This range provided the best separation in a reasonable time.

#### Internal standard selection

Different internal standards such as labetalol, aspirin, diazepam, furosemide, valsartan, mebeverine and linezolid were tried. None of the internal standards gave a well separated peak in a reasonable retention time. Labetalol and diazepam overlapped with AZE. Linezolid showed poor resolution with CTZ. Mebeverine eluted after 10.0 min with a distorted peak. The rest of the drugs eluted with the solvent front. So, the study was continued without utilizing internal standard.

#### Full factorial design

A full factorial design is a type of DOE (multivariate optimization). In this study, a 2^3^ ull factorial design (two levels and three independent factors) was applied for the optimization of the chromatographic separation. From the previous experiments, it was concluded that three independent factors had an impact on the chromatographic performance including: pH of the eluent, percentage of acetonitrile and flow rate. The 2^3^ full factorial design proposed eight experiments to determine the optimum conditions that gave the best values for the responses. The responses determined were: resolution between CTZ and AZE (R_s_), tailing factor of CTZ peak and tailing factor of AZE peak.

Minitab response optimizer was utilized to recognize the lower, target and upper values for the three responses (Table 1). Accordingly, the optimal conditions for the input values and desirability values were measured^1, 2^. The response optimizer measures composite desirability (D), which estimates whether the responses are within acceptable limits. This value is used to ensure that optimal conditions are reached and it ranges from zero to one. The optimal conditions can then be found by maximizing the composite desirability. The optimization plot in Minitab shows the effect of each factor on the responses or composite desirability and the interaction between them to give the optimal conditions^2^.

**Table 1:** Response optimization of 2^3^ full factorial design for RP-HPLC separation of CTZ/AZE mixture.

Parameters							Optimum conditions: pH = 5, %acetonitrile = 60%, flow rate = 1.2 ml min^−1^	
							Composite desirability (D) = 0.9427	
Responses	Goal	Lower	Target	Upper	Weight	Importance	Predicted responses	Individual desirability (d)
Rs	Maximize	2.76	4.24	4.24	1	1	4.24	1
Tailing of CTZ	Minimize	1.90	1.90	2.36	1	1	1.90	1
Tailing of AZE	Minimize	1.44	1.44	1.81	1	1	1.50	0.8378

##### Factors affecting R_s_ between CTZ and AZE

In accordance with the Pareto chart of the factor effects (Supplementary Fig. S1a online), the main effects plot (Supplementary Fig. S2a online) and the normal plot (Supplementary Fig. S3a online), %acetonitrile (B) has the strongest significant impact for a 95% confidence level and a positive proportion on Rs. Also, pH of the eluent (A) has a significant positive impact on it. According to the interaction plots (Supplementary Fig. S4a online), %acetonitrile has a positive correlation with R_s_ when it interacts with pH at low and high levels. The other parameters’ interactions have nearly no noticeable effect on R_s_.

##### Factors affecting tailing of CTZ

From the Pareto chart and interaction plots (Supplementary Figs. S1b and S4b online), the triple interaction (pH-acetonitrile-flow rate) has the highest impact on the tailing of CTZ peak. From the main effects plot for tailing of CTZ (Supplementary Fig. S2b online), flow rate (C) has the most potential negative impact as a single factor. Also, it has a negative influence when it interacts with pH or with %acetonitrile, whether at high or low levels for both factors.

##### Factors affecting tailing of AZE

As illustrated from the Pareto charts (Supplementary Fig. S1c online), the main effects plots (Supplementary Fig. S2c online) and the normal plot (Supplementary Fig. S3c online), flow rate (C) has the strongest negative impact on tailing of AZE. However, it is not significant at a 95% confidence level. Also, pH and %acetonitrile affect it negatively. The interaction plot (Supplementary Fig. S4c online) shows that %acetonitrile has an inverse impact on tailing of AZE when it interacts with pH at low and high levels. Additionally, flow rate has an inverse correlation with tailing of AZE when it interacts with pH or with %acetonitrile either at low or high levels for both factors.

The outcomes were statistically analyzed. Generally, the lower the coefficient of variation value, the better the reliability of the experiment. Coefficient of variation values were 17.98, 8.08 and 7.68 for R_s_, tailing of CTZ and tailing of AZE, respectively. Coefficient of determination (R^2^) is the proportion of the explained variance of the response. R^2^ values were 99.4%, 38.8% and 59.6% for R_s_, tailing of CTZ and tailing of AZE, respectively.

**Figure 3:**
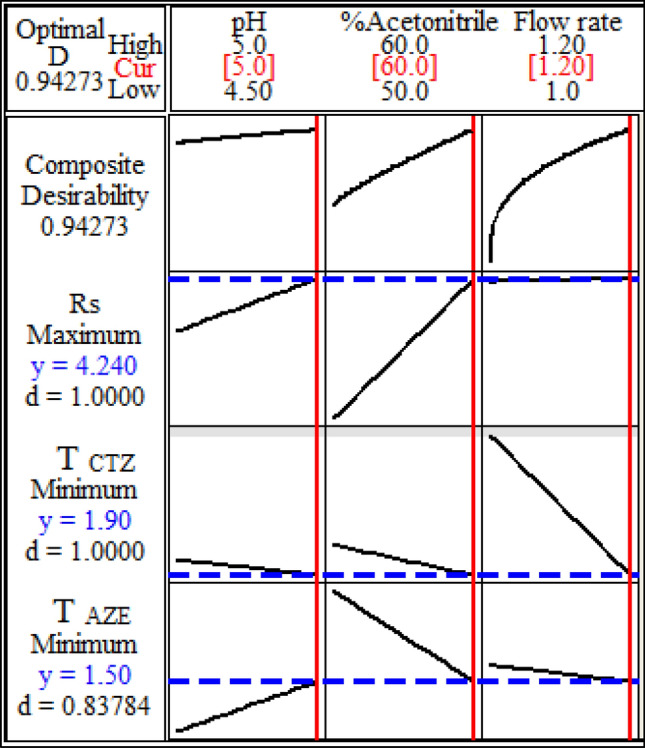
2^3^ full factorial design optimization plot for the chromatographic parameters.

**Figure 4:**
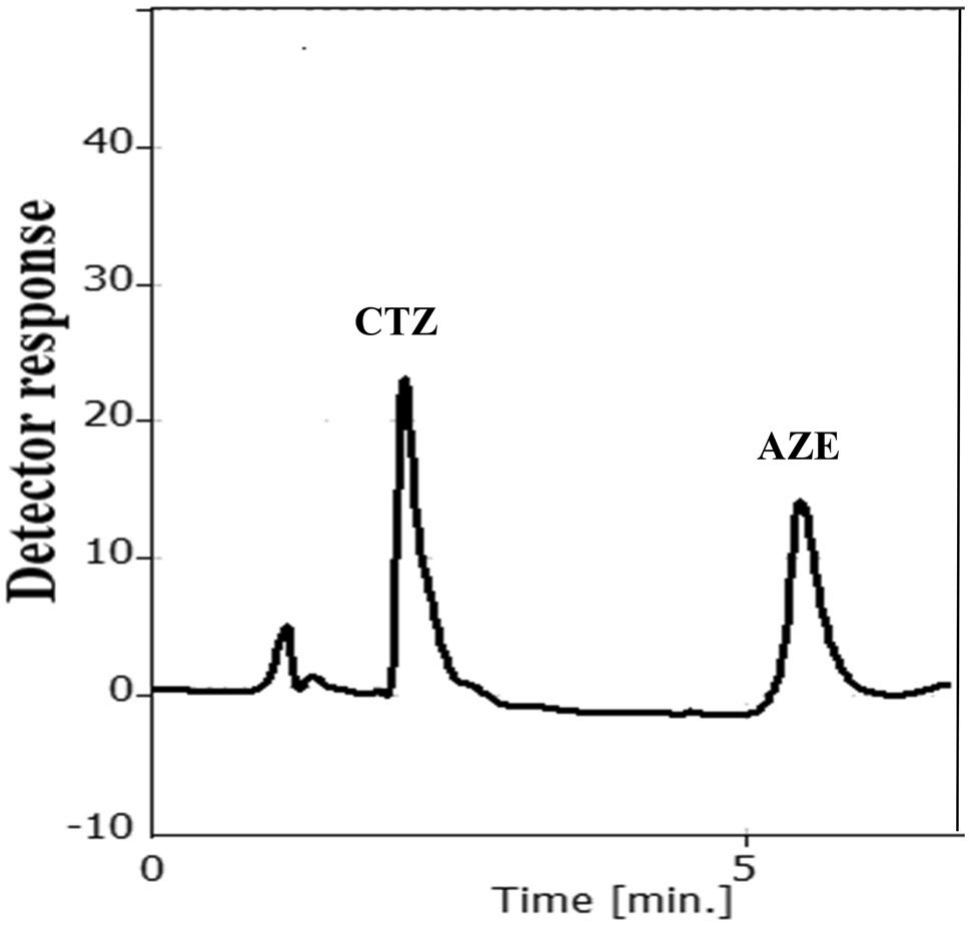
Chromatogram of a synthetic mixture of 15 µg mL^−1^ CTZ and 7 µg mL^−1^ AZE under the studied chromatographic conditions.

Consequently, the optimal mobile phase was acetonitrile: 0.3% TEA (pH 5) in the ratio of (60:40, v/v) and the flow rate was 1.2 mL min^−1^ depending on DOE (Fig. 3). Figure 4 presents the chromatogram of CTZ and AZE mixture.

## Validation of the proposed method

Validation of the analytical procedure was done in accordance with the ICH guidelines^51^ in order to ensure that it is appropriate for its intended use. The validation parameters were investigated as follows:

### Linearity and range

**Figure 5:**
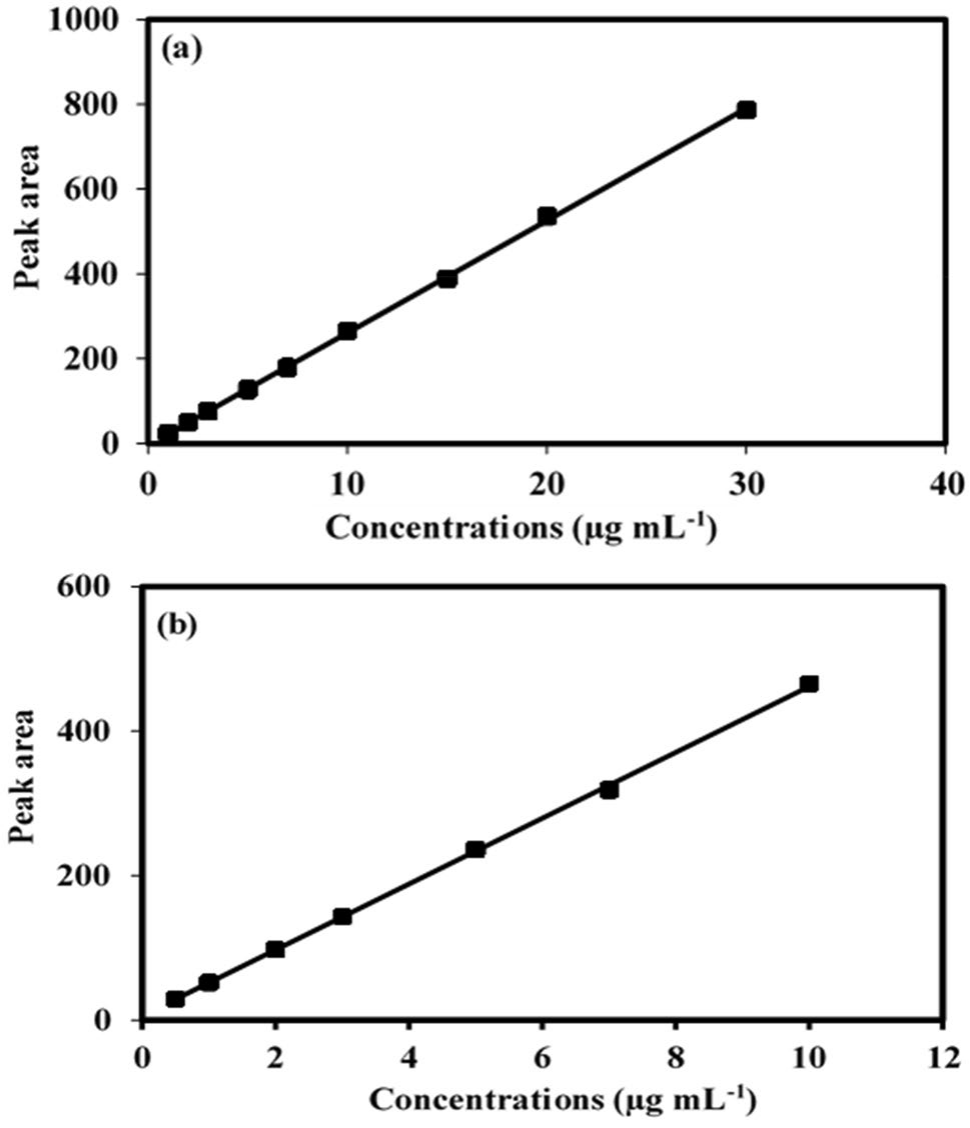
(**a**) Calibration graph of CTZ. (**b**) Calibration graph of AZE.

Calibration graphs for CTZ and AZE analysis were constructed between the peak area of each analyte and its concentration (µg mL^−1^) (Fig. 5). The designed approach was rectilinear over the concentration ranges of 1.0–30 for CTZ and 0.5–10 µg mL^−1^ for AZE. The following linear regression equations were derived from linear analyses:

$$\begin{gathered} {\text{Peak area}}\, = \, - {3}.{4454}\, + \,{26}.{\text{4582 C }}\left( {{\text{r}}\, = \,0.{9998}} \right){\text{ for CTZ,}} \hfill \\ {\text{Peak area}}\, = \,{6}.00{43}\, + \,{45}.{\text{5744 C }}\left( {{\text{r}}\, = \,0.{9998}} \right){\text{ for AZE}}{.} \hfill \\ \end{gathered}$$

where C is the analyte concentration (µg mL^−1^). Analytical data for the results are listed in Table 2.

**Table 2:** Analytical data for the adopted HPLC method.

Parameter	Cetirizine	Azelastine
Linearity range (µg mL^−1^)	1.0–30	0.5–10
Intercept (a)	− 3.4454	6.0043
Slope (b)	26.4582	45.5744
Correlation coefficient (r)	0.9998	0.9998
N	9	7
Standard deviation of residuals (S_y/x_)	4.9471	3.5797
Standard deviation of intercept (S_a_)	2.4889	2.1845
Standard deviation of slope (S_b_)	0.1804	0.4213
%Relative standard deviation (%RSD)	1.385	1.128
%Error	0.461	0.427
Limit of detection (LOD) (µg mL^−1^)	0.310	0.158
Limit of quantitation (LOQ) (µg mL^−1^)	0.941	0.479

### Limit of detection and limit of quantitation

LOD and LOQ were estimated as listed in Table 2 according to ICH criteria^51^, utilizing standard deviation of the intercept and the slope.

### Accuracy

The accuracy was confirmed by the acceptable percentage of recoveries from the quantitative measurement of CTZ and AZE bulk materials and synthetic mixtures. These data were compared to the data of the comparison synchronous spectrofluorimetric method^22^ and *t* and F values were calculated as listed in Table 3. These values point out the agreement of the data obtained by both methods^52^.

**Table 3:** Application of the studied HPLC method for the estimation of CTZ and AZE in raw materials.

Compound	Proposed method			Comparison method^22^
Cetirizine	Concentration taken (µg mL^−1^)	Concentration found (µg mL^−1^)	%found*	%found*
	1.0	1.012	101.20	101.33
	2.0	1.981	99.05	98.83
	3.0	2.991	99.70	100.30
	5.0	4.928	98.56	
	7.0	6.894	98.49	
	10.0	10.186	101.86	
	15.0	14.816	98.77	
	20.0	20.365	101.83	
	30.0	29.825	99.42	
$\bar{\text{X}} \pm {\text{S}}. {\text{D}}$			99.88 ± 1.38	100.15 ± 1.26
*t* **		0.31 (2.23)		
F **		1.20 (4.46)		
Azelastine	0.5	0.507	101.40	102.00
	1.0	1.006	100.60	99.14
	2.0	2.003	100.15	100.10
	3.0	3.006	100.20	
	5.0	5.045	100.90	
	7.0	6.853	97.90	
	10.0	10.077	100.77	
$\bar{\text{X}} \pm {\text{S}}. {\text{D}}$			100.27 ± 1.13	100.41 ± 1.46
*t* **		0.14 (2.31)		
F **		1.24 (5.14)		

*Average of three replicate estimations.

**The theoretical t and F values (*P* = 0.05) are between parentheses^52^.

### Precision

To test the intraday precision, three different concentrations of each analyte were analysed three times on the same day. The same concentrations were measured on three separate days to assess the interday precision. As illustrated in Supplementary Table S1 online, low SD, %RSD and %error values assure intraday and interday precision.

### Selectivity

Method selectivity was examined via the quantitative measurement of CTZ and AZE in aqueous humor without interference from its components. The high percentage of recoveries and low SD values for the analysis of both compounds in aqueous humor revealed the selectivity.

### Specificity

Method specificity was determined by testing the cited analytes in their commercial ophthalmic formulations and detecting interferences from dosage excipients. It was found that these excipients did not affect the assay performance. The results of the estimation of CTZ and AZE in raw materials and pharmaceutical formulations did not differ significantly.

### Robustness

The robustness was checked by deliberately varying the approach parameters, including pH (5.0 ± 0.1), acetonitrile ratio (60 ± 1%) and TEA concentration (0.3 ± 0.05). These variations did not have remarkable effect on the performance of the HPLC approach, indicating it is robust.

### System suitability

System suitability is an important parameter for ensuring that the resolution and reproducibility of the chromatographic procedure are adequate for the determination. Suitability parameters of the adopted approach were estimated as listed in Supplementary Table S2 online. The values are within the accepted ranges regarding USP^10^.

## Method applications

### Analysis of CTZ/AZE in laboratory prepared mixtures

The suggested approach was used to estimate CTZ and AZE in laboratory prepared mixtures. Supplementary Table S3 online shows acceptable percentage of recoveries.

### Analysis of CTZ/AZE in ophthalmic formulations

The developed method was effectively employed for the estimation of CTZ and AZE in their single eye drops. The data listed in Supplementary Table S4 online did not differ significantly from those attained from the comparison method^22^, as proved from *t* and F values^52^. No interference from excipients was detected.

### Analysis of CTZ/AZE in aqueous humor

**Figure 6:**
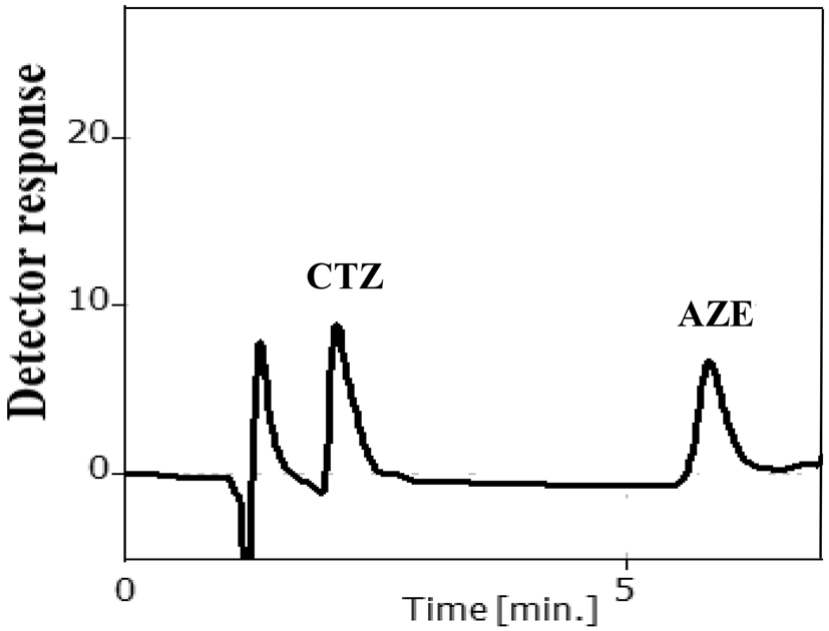
Chromatogram of spiked aqueous humor with 7 µg mL^−1^ CTZ and 3 µg mL^−1^ AZE under the studied chromatographic conditions.

Estimation of both medications concurrently in aqueous humor was adopted due to the high sensitivity and selectivity of the method (Fig. 6). In aqueous humor, CTZ and AZE had average absolute recoveries and %RSD of 100.93 ± 1.42 and 100.11 ± 1.55, respectively, as demonstrated in Supplementary Table S5 online.

## Conclusion

Eye drops must be administered at the correct dose to achieve optimal efficacy and minimal side effects for the patient. In severe ocular allergies, co-administration of CTZ and AZE eye drops may be recommended. As a result, it was critical to establish a simple, selective, accurate, precise and robust HPLC approach for the simultaneous analysis of CTZ and AZE in aqueous humor for therapeutic drug monitoring. Our HPLC–UV method was validated according to ICH guidelines and it shows improved resolution and sensitivity parameters and wider linearity ranges for both analytes. This is the first chromatographic procedure for concurrent separation of both drugs within a short analysis time (less than 6 min). The optimization of the chromatographic conditions was achieved by utilizing two level full factorial design, which reduces time and the number of trials. Furthermore, the approach was successfully applied in real life situations by estimating the cited drugs in their commercial eye drops and aqueous humor with good recoveries and without interference. The method is characterized by broad applicability, rapidity and adequate robustness. Accordingly, it can be easily applied in quality control laboratories and pharmacokinetic studies.

## Supplementary Information

Supplementary Information.

## Data Availability

The datasets generated and/or analyzed during the current study are available from the corresponding author on reasonable request (Walaa Nabil Abd-AlGhafar).

## References

[1] Saad AS, Ismail NS, Soliman M, Zaazaa HE (2016). Validated stability-indicating RP-HPLC method for simultaneous determination of clorsulon and ivermectin employing plackett-burman experimental design for robustness testing. J. AOAC Int..

[2] EL-Shorbagy HI, Elsebaei F, Hammad SF, El-Brashy AM (2019). Optimization and modeling of a green dual detected RP-HPLC method by UV and fluorescence detectors using two level full factorial design for simultaneous determination of sofosbuvir and ledipasvir: Application to average content and uniformity of dosage unit testing. Microchem. J..

[3] Candioti LV, De Zan MM, Cámara MS, Goicoechea HC (2014). Experimental design and multiple response optimization. using the desirability function in analytical methods development. Talanta.

[4] Miyazaki D (2020). Epidemiological aspects of allergic conjunctivitis. Allergol. Int..

[5] Ryder, EC, Benson, S 2021 Conjunctivitis. *StatPearls, *(StatPearls Publishing, Tampa).

[6] Jeremy Ono S, Abelson MB (2005). Allergic conjunctivitis: Update on pathophysiology and prospects for future treatment. J. Allergy Clin. Immunol..

[7] Bielory L, Friedlaender MH (2008). Allergic conjunctivitis. Immunol. Allergy Clin. North Am..

[8] Pharmacopoeia B. London: Her majesty's stationary office; Electronic version. 2022.

[9] Malhotra RP, Meier E, Torkildsen G, Gomes PJ, Jasek MC (2019). Safety of cetirizine ophthalmic solution 0.24% for the treatment of allergic conjunctivitis in adult and pediatric subjects. Clin. Ophthalmol..

[10] Pharmacopoeia 44 U. S., The National Formulary 39; The US Pharmacopoeial Convention; Rockville: MD. 2021.

[11] Ma M, Feng F, Sheng Y, Cui S, Liu H (2007). Development and evaluation of an efficient HPLC/MS/MS method for the simultaneous determination of pseudoephedrine and cetirizine in human plasma: Application to phase-I pharmacokinetic study. J. Chromatogr. B..

[12] Souri E, Hatami A, Ravari NS, Alvandifar F, Tehrani MB (2013). Validating a stability indicating HPLC method for kinetic study of cetirizine degradation in acidic and oxidative conditions. Iran. J. Pharm. Res..

[13] Aly F, EL-Enany N, Elmansi H, Nabil AA (2017). Validated reversed phase hplc method for simultaneous determination of the antihistaminic cetirizine and beta2-adrenergic agonist salbutamol in their co-formulated tablets. SM Anal. Bioanal. Technique.

[14] Shamshad H, Naz A, Mirza AZ (2021). Reverse phase HPLC method for the simultaneous determination of cetirizine, verapamil/Diltiazem and amlodipine. Anal. Bioanal. Chem. Res..

[15] Shamshad H, Mirza AZ (2021). Application of RP-HPLC method for the simultaneous determination of cetirizine in the presence of quinolones. Future J. Pharm. Sci..

[16] Shamshad H, Sayqal A, Zeb Z, Mirza AZ (2021). Simultaneous determination of chloroquine and pyrimethamine with cetirizine in an active form and human serum by RP-HPLC. J. Chromatogr. Sci..

[17] Uysal UD, Tunçel M (2006). Validated capillary electrophoresis study for the determination of cetirizine in pharmaceutical forms. J. Liq. Chromatogr. Relat. Technol..

[18] Javid FS, Shafaat A, Zarghi A (2014). Determination of cetirizine and its impurities in bulk and tablet formulation using a validated capillary zone electrophoretic method. J. Anal. Chem..

[19] Ibrahim F, El-Din MS, Eid M, Wahba MEK (2011). Spectrofluorimetric determination of some H1 receptor antagonist drugs in pharmaceutical formulations and biological fluids. Int. J. Pharm. Sci. Res..

[20] Wei XL, Gong Q, Wang LS, Liao Y (2011). Determination of cetirizine dihydrochloride by anti-fluorescence quenching on rhodamine B-Sodium tetraphenylborate system. Guang Pu Xue Yu Guang Pu Fen Xi.

[21] El-Din MKS, Ibrahim F, Eid MI, Wahba MEK (2012). Validated spectroflurimetric determination of some H1 receptor antagonist drugs in pharmaceutical preparations through charge transfer complexation. J. Fluoresc..

[22] Abd-AlGhafar WN, Aly FA, Sheribah ZA, Saad S (2022). Synchronous fluorescence as a green and selective method for the simultaneous determination of cetirizine and azelastine in aqueous humor. J. Fluoresc..

[23] El-Kommos ME, El-Gizawy SM, Atia NN, Hosny NM (2015). Determination of some non-sedating antihistamines via their native fluorescence and derivation of some quantitative fluorescence intensity-structure relationships. J. Fluoresc..

[24] Pourghazi K, Khoshhesab ZM, Golpayeganizadeh A, Shapouri MR, Afrouzi H (2011). Spectrophotometric determination of cetirizine and montelukast in prepared formulations. Int. J. Pharm. Pharm. Sci..

[25] El-Didamony AM, Ramadan GM (2020). Charge-transfer interaction between antihistamine antiallergic drugs, diphenhydramine, fexofenadine, cetirizine and two π-acceptors in pharmaceutical forms. SN Appl. Sci..

[26] Lambiase A, Micera A, Bonini S (2009). Multiple action agents and the eye: Do they really stabilize mast cells?. Curr. Opin. Allergy Clin. Immunol..

[27] El-Shaheny RN, Yamada K (2014). Stability study of the antihistamine drug azelastine HCl along with a kinetic investigation and the identification of new degradation products. Anal. Sci..

[28] Hassouna M, Abdelrahman M, Abdelfatah M (2017). Simultaneous determination of azelastine hydrochloride and benzalkonium chloride by RP-HPLC method in their ophthalmic solution. Anal. Sci..

[29] Patel S, Pasha TY (2018). Stability-indicating high-performance liquid chromatography method for determination of antihistamine drug azelastine. Asian J. Pharm. Clin. Res..

[30] El-Masry AA, Hammouda MEA, El-Wasseef DR, El-Ashry SM (2019). Eco-friendly green liquid chromatographic determination of azelastine in the presence of its degradation products: Applications to degradation kinetics. J. AOAC Int..

[31] El-Masry AA, Hammouda ME, El-Wasseef DR, El-Ashry SM (2020). Eco-friendly green liquid chromatographic separations of a novel combination of azelastine and fluticasone in the presence of their pharmaceutical dosage form additives. Curr. Anal. Chem..

[32] Salama NN, Abdel-Razeq SA, Abdel-Atty S, El-Kosy N (2014). Development and validation of densitometry TLC stability indicating method for quantitative determination of azelastine hydrochloride and emedastine difumarate in their drug products. J. Pharm. Res. Int..

[33] Patel KG, Patel SKG, Shah PA, Tandel DB, Gandhi TR (2020). Development and validation of HPTLC method along with forced degradation study for the simultaneous estimation of azelastine hydrochloride and fluticasone propionate in nasal spray formulation using design of experiment approach. Indian J. Pharm. Educ. Res..

[34] Abdelwahab NS, Farid NF, Elagawany M, Abdelmomen EH (2018). Efficient UPLC and CE methods for the simultaneous determination of azelastine hydrochloride and its genotoxic impurity. Biomed. Chromatogr..

[35] Abdel-Razeq SA, Foaud MM, Salama NN, Abdel-Atty S, El- Kosy N (2011). Voltammetric determination of azelastine-HCl and emedastine dirumarate in micellar solution at glassy carbon and carbon paste electrodes. Sens. Electroanal..

[36] Badran OM, Salem MY, Kelani KM (2013). Application of membrane selective electrodes for the determination of azelastine hydrochloride in the presence of its alkaline degradant in eye drops and plasma. J. Anal. Bioanal. Electrochem..

[37] El-Masry AA, El-Wasseef DR, Eid M, Shehata IA, Zeid AM (2021). Quantitative proton nuclear magnetic resonance method for simultaneous analysis of fluticasone propionate and azelastine hydrochloride in nasal spray formulation. R. Soc. Open. Sci..

[38] El-Masry AA, Hammouda MFA, El-Wasseef DR, El-Ashry SM (2017). Validated sensitive spectrofluorimetric method for determination of antihistaminic drug azelastine HCl in pure form and in pharmaceutical dosage forms: Application to stability study. Luminescence.

[39] Ragab MAA, El-Kimary EI (2018). Investigation of the spectrofluorimetric behavior of azelastine and nepafenac: Determination in ophthalmic dosage forms. Spectrochim. Acta A..

[40] Shekhar S, Bali A (2021). Spectrofluorimetric method for the determination of azelastine hydrochloride in bulk and nasal formulations. J. Appl. Spectrosc..

[41] Gouda AA, El Sheikh R, El Saied H (2015). Extractive spectrophotometric determination of azelastine hydrochloride in pure form and pharmaceutical formulations. Can. Chem. Trans..

[42] Hassouna ME, Abdelrahman MM, Mohamed MA (2017). Determination of azelastine hydrochloride and benzalkonium chloride in their ophthalmic solution by different spectrophotometric methods. World J. Appl. Chem..

[43] El-Masry AA, Hammouda MEA, El-Wasseef DR, El-Ashry SM (2018). Validated spectroscopic methods for determination of anti-histaminic drug azelastine in pure form: Analytical application for quality control of its pharmaceutical preparations. Spectrochim. Acta A..

[44] Castillo, M., Scott, N. W., Mustafa, M. Z., Mustafa, M. S. & Azuara‐Blanco, A. Toxpical antihistamines and mast cell stabilisers for treating seasonal and perennial allergic conjunctivitis. Cochrane Database Syst. Re. **6.** (2015). 10.1002/14651858.CD009566.pub2 PMC10616535 26028608

[45] Rozet E, Lebrun P, Hubert P, Debrus B, Boulanger B (2013). Design Spaces for analytical methods. TrAC Trends Anal. Chem..

[46] Pietrowska K (2018). Analysis of pharmaceuticals and small molecules in aqueous humor. J. Pharm. Biomed. Anal..

[47] Nikolin B, Imamović B, Medanhodzić-Vuk S, Sober M (2004). High perfomance liquid chromatography in pharmaceutical analyses. Bosn. J. Basic Med. Sci..

[48] Macri A (2015). An artificial aqueous humor as a standard matrix to assess drug concentration in the anterior chamber by high performance liquid chromatography methods. Clin. Lab..

[49] Moffat AC, Osselton MD, Widdop B, Watts J (2011). Clarke’s analysis of drugs and poisons.

[50] Mendez A, Bosch E, Roses M, Neue UD (2003). Comparison of the acidity of residual silanol groups in several liquid chromatography columns. J. Chromatogr. A..

[51] Guideline IHT (2005). Validation of analytical procedures: Text and methodology. Q2 (R1).

[52] Miller JN, Miller JC (2008). Statistics and chemometrics for aanalytical chemistry.

