# Supplementary material for: Factorial design-assisted reverse phase HPLC–UV approach for the concurrent estimation of cetirizine and azelastine in aqueous humor
Source: Sci Rep. 2022 Dec 27;12:22435. doi: 10.1038/s41598-022-26774-y (PMC9794793; doi:10.1038/s41598-022-26774-y)
Supplement: Supplementary file 1 — Supplementary Information. [file 41598_2022_26774_MOESM1_ESM.pdf]

Factorial design-assisted reverse phase HPLC-UV approach for the concurrent estimation of cetirizine and azelastine in aqueous humor

Walaa Nabil Abd-AlGhafar*, Fatma Ahmed Aly, Zeinab Awad Sheribah, Samar Saad

Mansoura University, Faculty of Pharmacy, Pharmaceutical Analytical Chemistry Department, 35516 Mansoura, Egypt

* Corresponding author

Walaa Nabil Abd-AlGhafar

Tel.: ++20502352714 Fax: ++20502247496 e-mail: walaanabil246@mans.edu.eg

Supplementary Table S1. Precision data for the analysis of CTZ and AZE by the suggested method.

		Intraday precision			Interday precision		
Concentration ($\mu g\ mL^{-1}$)		Mean ± S.D.	%RSD	%Error	Mean ± S.D.	%RSD	%Error
**Cetirizine**	3.0	101.14 ± 1.34	1.33	0.77	100.34 ± 0.79	0.79	0.45
	10	99.70 ± 1.88	1.88	1.09	101.68 ± 0.16	0.16	0.09
	20	101.72 ± 0.85	0.84	0.49	101.95 ± 0.29	0.28	0.16
**Azelastine**	1.0	101.25 ± 0.72	0.71	0.41	100.89 ± 0.27	0.27	0.16
	3.0	98.76 ± 1.24	1.26	0.73	99.37 ± 1.19	1.20	0.69
	7.0	98.63 ± 0.74	0.75	0.43	98.65 ± 0.08	0.08	0.04

Supplementary Table S2. System suitability parameters of the studied HPLC approach.

Parameter	CTZ	AZE
**Number of theoretical plates (NTP)**	852	1697
**Capacity factor (k`)**	0.98	3.83
**Resolution (R_s)**	4.30	
**Tailing factor (T)**	1.90	1.45

$$NTP = 5.54 \left(\frac{t_R}{W_{h/2}}\right)^2$$

$$T = W_{0.05}/2f$$

$$k` = \frac{t_R - t_m}{t_m}$$

$$R_s = \frac{2\Delta t_R}{W_1 + W_2}$$

Supplementary Table S3. Analytical data for the determination of CTZ and AZE in their synthetic mixtures by the suggested HPLC method.

Parameter	Proposed method						Comparison method[22]	
	Concentration taken (μg mL^{-1})		Concentration found (μg mL^{-1})		%found*		%found*	
	CTZ	AZE	CTZ	AZE	CTZ	AZE	CTZ	AZE
	1.0	1.0	1.007	1.018	100.71	101.80	99.13	101.60
	10	1.0	10.161	0.981	101.61	98.07	98.69	98.33
	10	3.0	9.806	2.942	98.06	98.08	101.13	100.20
	5.0	5.0	5.081	4.902	101.63	98.04		
	15	7.0	14.632	6.824	97.55	97.48		
**$\bar{X}$ ± S.D.**					99.91 ± 1.97	98.69 ± 1.75	99.65 ± 1.30	100.04 ± 1.64
t**[**]**					0.22 (2.45)	1.10 (2.45)		
**F**[**]**					2.30 (6.94)	1.14 (6.94)		

* Average of three replicate estimations.

** The theoretical t and F values (P = 0.05) are between parentheses[52].

Supplementary Table S4. Analytical data for the determination of CTZ and AZE in single ophthalmic formulations by the suggested HPLC method.

Ophthalmic formulations	Proposed method			Comparison method[22]
	Concentration taken (μg mL^{-1})	Concentration found (μg mL^{-1})	%found*	%found*
**Cetirizine® 1%**	2.0	2.015	100.74	101.80
	5.0	4.902	98.04	98.70
	7.0	6.863	98.05	100.20
	20	20.392	101.96	
**$\bar{X}$ ± S.D.**			99.70 ± 1.97	100.23 ± 1.55
t**			0.40 (2.57)	
**F****			1.62 (9.55)	
**Azelast® 0.05%**	1.0	1.013	101.27	99.40
	2.0	1.992	99.61	100.71
	5.0	5.035	100.71	99.80
	10	10.203	102.03	
**$\bar{X}$ ± S.D.**			100.91 ± 1.02	99.97 ± 0.67
t**			1.47 (2.57)	
**F****			2.32 (9.55)	

* Average of three replicate estimations.

** The theoretical t and F values (P = 0.05) are between parentheses[52].

Supplementary Table S5. Analytical data for the determination of CTZ and AZE in spiked aqueous humor by the suggested HPLC method.

**Parameter**	**Concentration taken (µg mL^{-1})**	**Concentration taken (µg mL^{-1})**	**Concentration found (µg mL^{-1})**	**Concentration found (µg mL^{-1})**	**%found**	**%found**
	**CTZ**	**AZE**	**CTZ**	**AZE**	**CTZ**	**AZE**
	10	0.5	10.155	0.489	101.55	97.84
	20	1.0	20.426	1.013	102.13	101.27
	10	2.0	10.118	2.036	101.18	101.81
	3.0	3.0	3.040	2.999	101.35	99.97
	7.0	3.0	6.892	2.990	98.46	99.66
**$\bar{X}$ ± S.D.**					100.93 ± 1.43	100.11 ± 1.55
**%RSD**					1.42	1.55
**%Error**					0.63	0.69

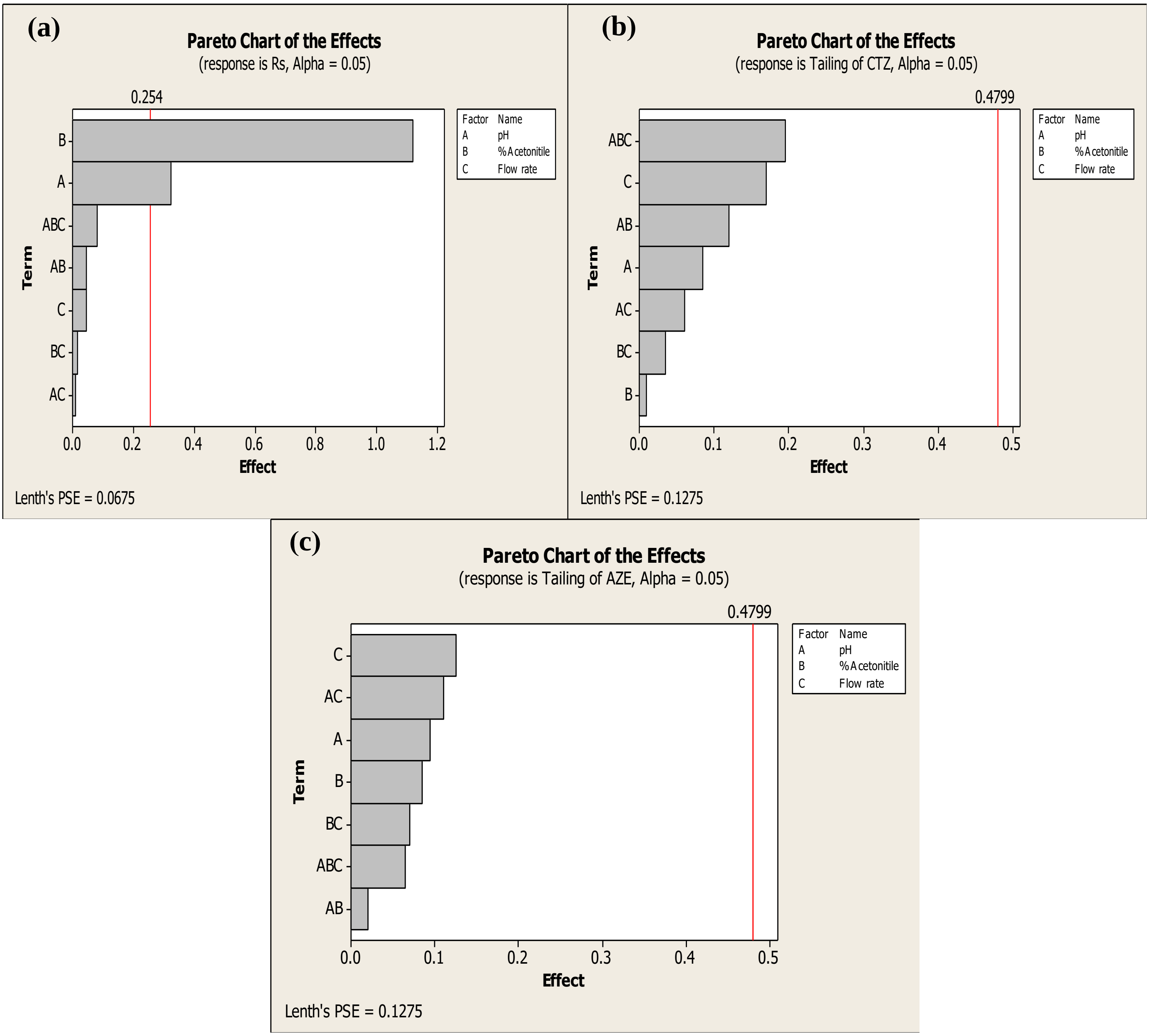

Supplementary Fig. S1. 2^3 full factorial design Pareto charts of the effects on the chromatographic responses at alpha = 0.05.

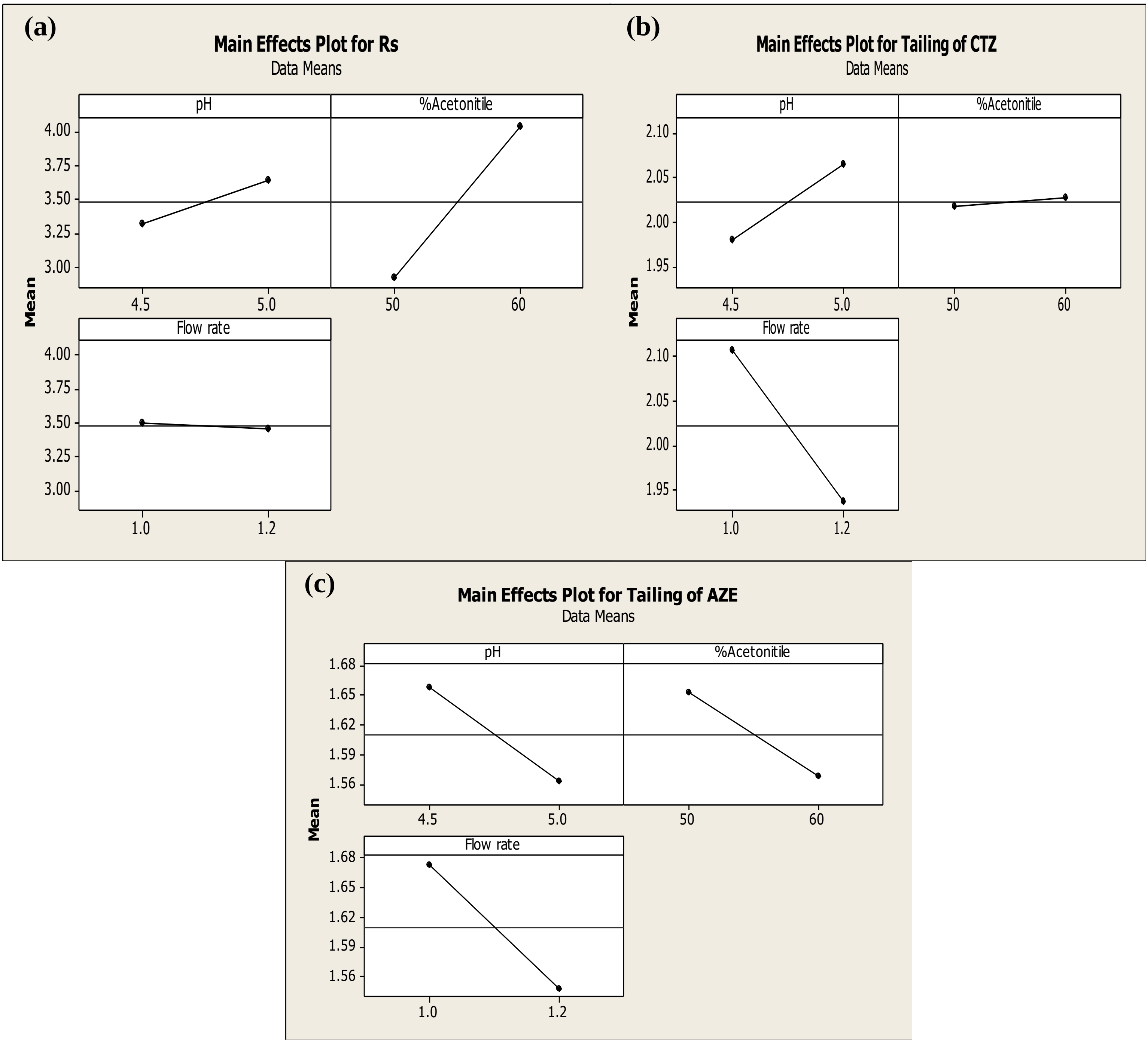

Supplementary Fig. S2. 2^3 full factorial design main effect plots for chromatographic responses by data means type.

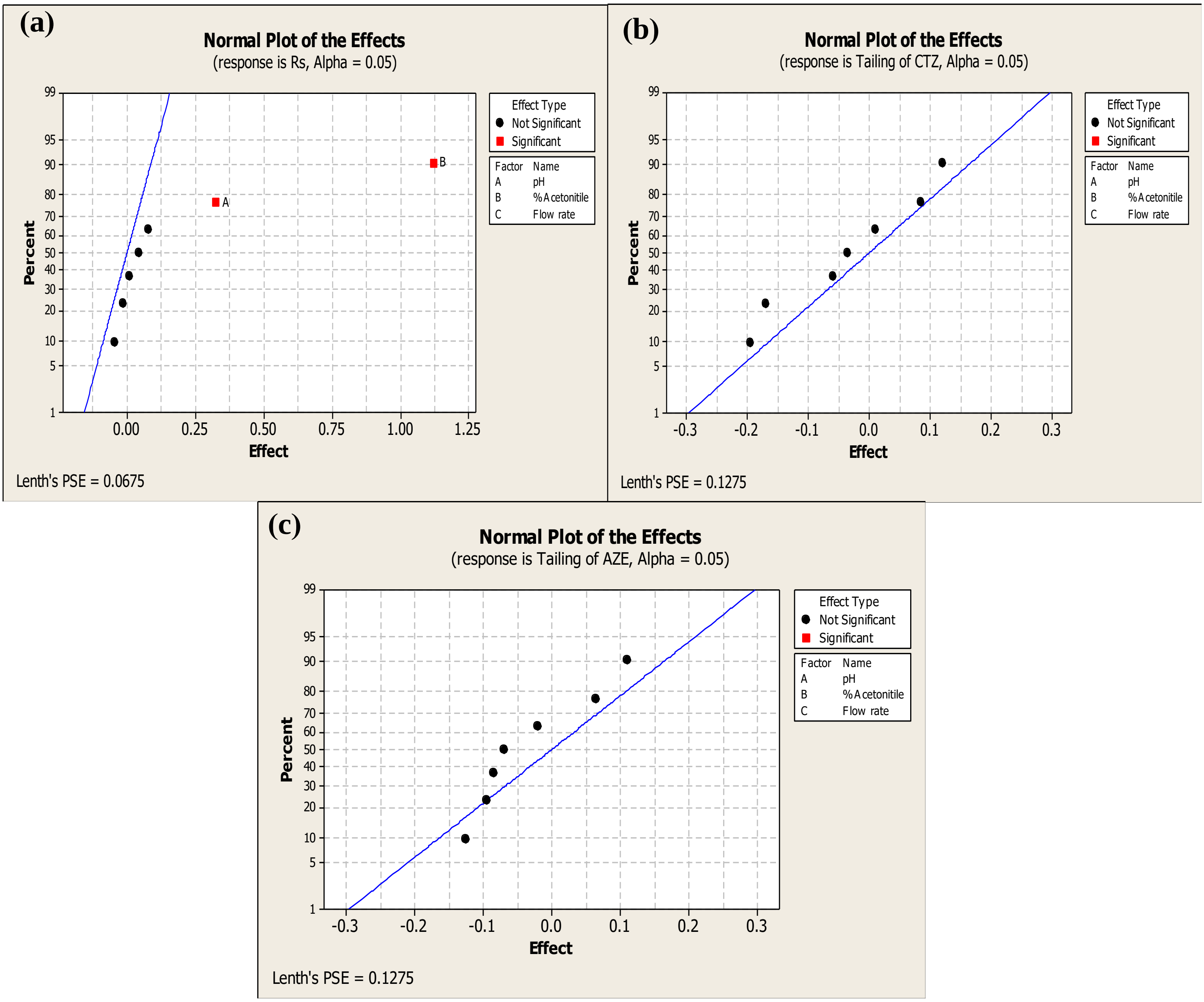

Supplementary Fig. S3. 2^3 full factorial design normal plots of the effects on the chromatographic responses at alpha = 0.05.

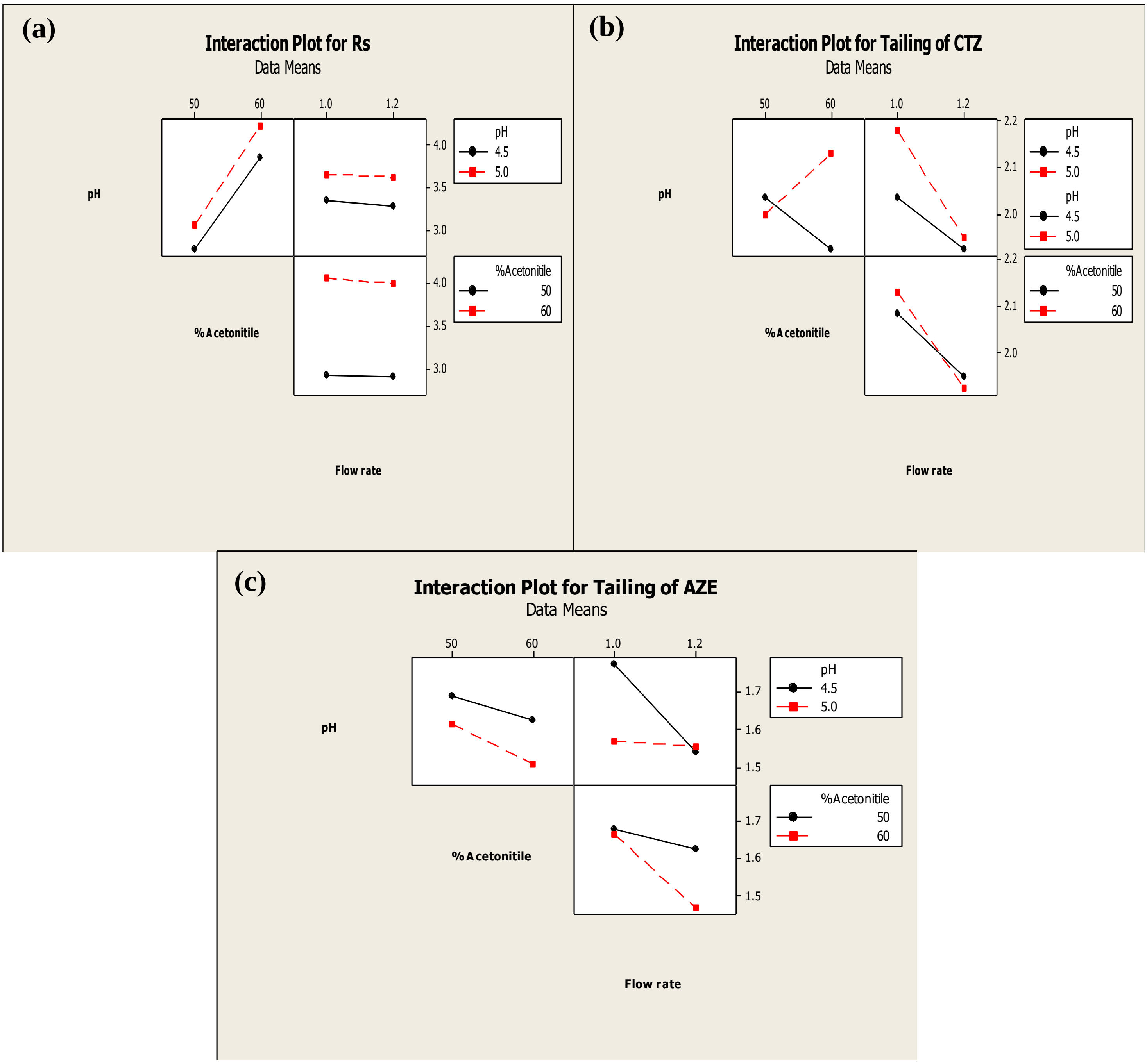

Supplementary Fig. S4. 2^3 full factorial design interaction plots for chromatographic responses by data means type.
